# Effectiveness of Lower Extremity Exercise and Hydration on Orthostatic Hypotension Among Patients With Schizophrenia in Psychiatric Hospital: A Quasiexperimental Study

**DOI:** 10.1155/nrp/7518475

**Published:** 2026-09-28

**Authors:** Sari Narulita, Doreen Sumpat, Richard Avoi, Nicholas Pang Tze Ping, Sumarni Situmorang, Mochammad Udji Priyatna, Neng Esti Winahayu, Dheni Wahyudi, Dwinara Febrianti, Ellya Qolina

**Affiliations:** ^1^ Faculty Nursing and Midwifery, Univesity of Binawan, Jakarta, Indonesia; ^2^ Faculty of Medicine and Health Science, University of Malaysia Sabah, Kota Kinabalu, Malaysia; ^3^ Marzoeki Mahdi Hospital, Bogor, Indonesia; ^4^ Soeharto Heerdjan Hospital, Jakarta, Indonesia; ^5^ Duren Sawit Regional Special Hospital, Jakarta, Indonesia; ^6^ Faculty of Health Science, University of Thamrin, Jakarta, Indonesia; ^7^ Faculty of Health Science, University of Cendikia Abditama, Tangerang, Indonesia

**Keywords:** antipsychotic, hydration, lower extremity exercise, orthostatic hypotension, patient safety, schizophrenia

## Abstract

**Background:**

Orthostatic hypotension (OH) is a common adverse effect of antipsychotic therapy and represents a significant patient safety concern in psychiatric nursing practice due to its association with falls and cardiovascular instability. Nurse‐led, nonpharmacological interventions may offer feasible strategies to manage this condition in inpatient mental health settings. Although nonpharmacological interventions such as hydration and lower limb exercise have shown effectiveness in managing OH in older adults and neurological populations, evidence in psychiatric inpatients remains limited. Patients with schizophrenia receiving antipsychotics present unique physiological and behavioral challenges, including medication‐induced autonomic dysfunction, low physical activity, and inconsistent fluid intake. To date, no quasiexperimental study has evaluated the comparative and combined effects of lower extremity exercise and hydration in this specific population.

**Aim:**

This study aimed to compare the effectiveness of nurse‐led lower extremity exercise, water consumption, and their combination in reducing OH among hospitalized patients with schizophrenia receiving antipsychotic treatment.

**Methods:**

This quasiexperimental study was conducted in a psychiatric hospital. A total of 180 patients diagnosed with schizophrenia were enrolled and allocated into four equal groups (*n* = 45 per group): control, lower extremity exercise, water consumption, and combined intervention groups. Blood pressure was measured in the supine position and at 1, 3, 5, and 10 min after standing. Data were analyzed using repeated measures ANOVA.

**Results:**

The results of the repeated measures ANOVA revealed a statistically significant difference in systolic blood pressure (SBP) change between intervention groups (*p* = 0.006). The lower extremity exercise group demonstrated the highest estimated marginal mean for SBP improvement, and Bonferroni‐adjusted pairwise comparisons showed a significant difference only between the lower extremity exercise and control groups. Repeated measures ANOVA demonstrated a significant difference in diastolic blood pressure (DBP) between intervention groups (*p* = 0.010). The combined intervention demonstrated the highest estimated marginal mean for DBP, although Bonferroni‐adjusted pairwise comparisons showed no statistically significant differences between intervention groups.

**Conclusion:**

This study provides evidence that nursing‐led, nonpharmacological interventions, specifically lower extremity exercise and water consumption, are effective in reducing OH among hospitalized patients with schizophrenia receiving antipsychotic treatment. Lower extremity exercise demonstrated the highest estimated marginal mean for SBP improvement, whereas the combined intervention showed the highest estimated marginal mean for DBP. These findings support the implementation of simple, evidence‐based nursing interventions to improve hemodynamic stability and enhance patient safety in psychiatric inpatient care.

**Trial Registration:** International Standard Randomized Controlled Trial Number: ISRCTN10651820

## 1. Introduction

Orthostatic hypotension (OH) is defined as a significant drop in blood pressure upon standing, specifically a decrease in systolic blood pressure (SBP) of at least 20 mmHg or diastolic blood pressure (DBP) of at least 10 mmHg within three minutes of standing [1]. This condition is particularly concerning to schizophrenia patients with OH; this condition may lead to dizziness, syncope, falls, cardiovascular instability, and functional impairment. If left unrecognized, OH may contribute to fall‐related injuries, cardiovascular complications, prolonged hospitalization, increased healthcare utilization, and poorer clinical outcomes. These consequences are particularly relevant in psychiatric settings, where impaired mobility, antipsychotic treatment, and reduced treatment adherence may further delay recovery and compromise patient safety. Therefore, early recognition and effective management of OH remain important priorities in psychiatric nursing practice.

Schizophrenia is a chronic psychiatric disorder characterized by disturbances in thought, perception, and behavior, often requiring long‐term antipsychotic therapy. While effective in managing psychotic symptoms, antipsychotic medications are associated with various side effects, including OH, which can increase the risk of falls and syncope, and negatively impact patient adherence to treatment [2–6].

In psychiatric inpatient settings, OH remains underrecognized despite its clinical significance. Previous studies reported that 9.43% of patients receiving antipsychotic treatment experienced OH, while another study reported a prevalence of 32.67% among hospitalized psychiatric patients [7, 8]. Despite this high prevalence, management strategies for OH in psychiatric populations remain predominantly pharmacological, including dose reduction or switching antipsychotic agents. However, these approaches are often clinically challenging due to symptom severity, relapse risk, and limited therapeutic alternatives [5].

Non‐pharmacological interventions, such as lower extremity exercises and water consumption, have been proposed as effective strategies to mitigate OH [9, 10]. Lower extremity exercises can enhance venous return and improve autonomic regulation, while water intake can expand plasma volume, both contributing to the stabilization of blood pressure upon standing [6, 11–14].

However, most previous studies evaluating nonpharmacological interventions for OH have focused on older adults, neurological disorders, or postoperative populations, with limited evidence available in psychiatric inpatients. Although several nonpharmacological strategies have been recommended for OH, including compression stockings, abdominal binders, salt supplementation, and physical counter‐maneuvers, many of these interventions present practical limitations in psychiatric inpatient settings. Compression garments may be poorly tolerated, require sustained patient adherence, and present practical and safety challenges in individuals with acute psychosis, agitation, or impaired cooperation. Likewise, interventions requiring complex behavioral modifications or prolonged supervision are often difficult to implement consistently in routine psychiatric nursing care. In contrast, lower extremity exercise and water consumption are simple, inexpensive, nurse‐led interventions that can be safely integrated into daily ward routines while directly targeting two key physiological mechanisms of OH: enhancement of the skeletal muscle pump to improve venous return and expansion of intravascular plasma volume. These characteristics make them particularly suitable for hospitalized patients with schizophrenia receiving antipsychotic treatment, who frequently experience autonomic dysfunction, sedentary behavior, and inadequate fluid intake. Furthermore, previous intervention studies generally evaluated a single intervention strategy, lacked comprehensive comparison groups, and primarily assessed immediate or short‐term blood pressure responses rather than repeated daily measurements. Despite this strong physiological rationale, no previous quasiexperimental study has directly compared the effectiveness of lower extremity exercise, water consumption, and their combination in this specific psychiatric population.

To address this gap, this quasi experimental study aimed to compare the effectiveness of lower extremity exercise, water consumption, and their combination in reducing OH among hospitalized patients with schizophrenia receiving antipsychotic treatment. This study is expected to provide evidence supporting practical, low‐cost, nurse‐led interventions to improve patient safety and facilitate the integration of nonpharmacological management into routine psychiatric nursing care.

## 2. Methods

### 2.1. Study Design and Setting

This study employed a quasiexperimental design with four groups: a control group, a combined lower extremity exercise and water consumption group, a lower extremity exercise group, and a water consumption group. The study was conducted in a psychiatric hospital inpatient setting to evaluate the effectiveness of nonpharmacological interventions in reducing OH among patients with schizophrenia receiving antipsychotic treatment. Reporting is in accordance with the Transparent Reporting of Evaluations with Non‐randomized Designs (TREND) statement for quasiexperimental studies.

The study commenced with a screening phase (Day 0) targeting patients diagnosed with schizophrenia who were suspected of experiencing OH. During this phase, all psychiatric inpatients underwent orthostatic blood pressure measurement to confirm the presence of OH, defined as a sustained drop in SBP of ≥ 20 mmHg or DBP of ≥ 10 mmHg defined within 3 min of standing; additional measurements at 5 and 10 min were performed for monitoring.

Patients who met the criteria for OH were subsequently assessed for eligibility through inclusion and exclusion criteria. Those who fulfilled all requirements were then allocated into intervention groups, ensuring that each group comprised individuals with clinically confirmed OH. Inclusion criteria were as follows: (1) diagnosed with schizophrenia, (2) undergoing antipsychotic treatment, (3) identified as having OH based on blood pressure measurements, and (4) in a calm, nonaggressive state. Exclusion criteria included (1) hemoglobin levels < 10 g/dL, (2) estimated glomerular filtration rate (eGFR) < 30%, (3) New York Heart Association (NYHA) Class III or IV heart failure, (4) undergoing dialysis, (5) violent behavior, (6) recent lower limb orthopedic surgery, and (7) a history of uncontrolled hypertension. Participants who met the eligibility criteria were allocated into intervention groups using nonrandomized ward‐based assignment according to clinical and operational feasibility within the psychiatric inpatient setting. Separate wards were used for each intervention group to minimize contamination between interventions and maintain intervention consistency.

Following group allocation, the assigned intervention was administered once daily for five consecutive days (Day 1 to Day 5). Participants in the lower extremity exercise group performed one supervised exercise session each day, whereas participants in the hydration group received the prescribed water consumption protocol daily. Participants in the combined intervention group received both interventions sequentially each day. Orthostatic blood pressure measurements were performed daily throughout the intervention period using the same standardized procedure, with measurements obtained in the supine position and at 1, 3, 5, and 10 min after standing. Baseline orthostatic blood pressure was measured on Day 0 before the intervention commenced, and follow‐up measurements were repeated each day from Day 1 through Day 5 to evaluate changes in blood pressure over time. Figure 1 illustrates the overall study timeline and intervention schedule.

**FIGURE 1:**
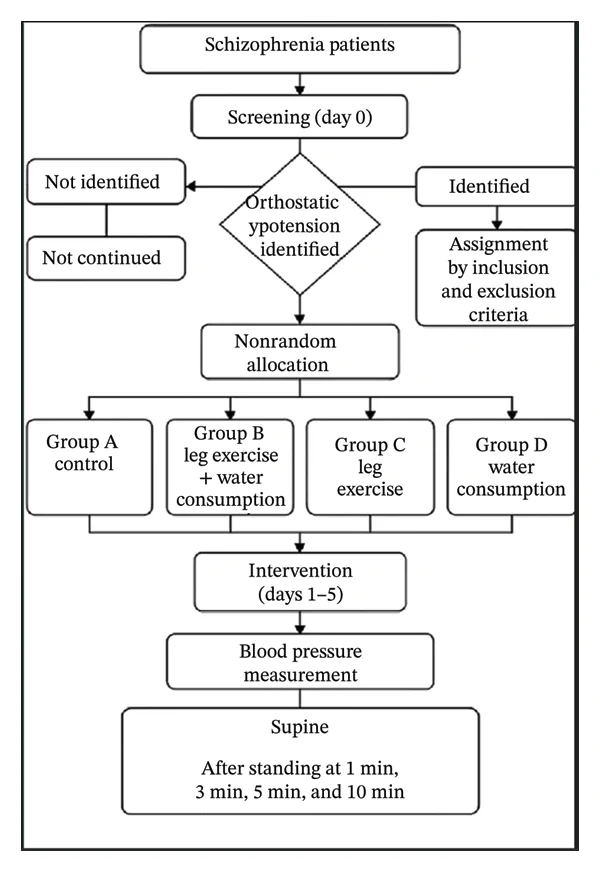
Research flowchart of the quasiexperimental design.

### 2.2. Participants

Sample size was calculated using G∗Power for repeated measures ANOVA, assuming an effect size of 0.25, an alpha level of 0.05, a statistical power of 0.80, four intervention groups, and five repeated measurements. The minimum required sample size was 160 participants. To ensure that each intervention group achieved the planned sample size after participant withdrawal or incomplete follow‐up, recruitment continued until 45 participants with complete outcome data were available in each group, resulting in a final analytical sample of 180 participants. The final analysis included 180 participants distributed across the four intervention groups, all of whom completed the five‐day intervention protocol and had complete blood pressure measurement data at all scheduled assessment time points.

### 2.3. Ethical Consideration

Ethical approval for this study was obtained from the Ethics Committee of the Faculty of Medicine and Health Sciences, Atma Jaya Catholic University of Indonesia (approval no. 0006P/III/PPPE.PM.10.05/06/2023). Written informed consent was obtained from all participants or their legally authorized representatives prior to enrollment. Participants were informed that participation was voluntary and that they could withdraw from the study at any time without affecting their treatment. All personal information was kept confidential, and only aggregated data were used for analysis and reporting.

### 2.4. Intervention Procedures

Interventions were administered by nurses with clinical experience in psychiatric inpatient care and who had received prior protocol training from the principal investigator. The exercise intervention was conducted individually to ensure safety, protocol adherence, and accurate blood pressure monitoring.

Group A (control): The control group received standard ward care, including medication administration, routine vital sign monitoring, assistance with activities of daily living, scheduled psychiatric evaluation, and general nursing observation. No structured hydration protocol or lower extremity exercise intervention was administered.

Group B (lower extremity exercise + water consumption): Participants received a combination of lower extremity exercise and water consumption interventions.

Water consumption: Participants in the hydration intervention group were instructed to consume at least 2000 mL of water within a 24‐h period. Water intake was standardized using identical 2‐L bottles with volume indicators and participant identification labels. Adequacy of intake was confirmed by direct monitoring and documentation by ward nurses before proceeding with lower extremity exercise intervention and orthostatic blood pressure assessment. In the combined intervention group, hydration adequacy was confirmed before the lower extremity exercise session was initiated.

Lower extremity exercise: Before starting the exercise, participants were instructed to lie in a supine position for five minutes, and their blood pressure was measured and recorded in that position. The exercise was conducted in a supine position using 3‐kg ankle weights (1.5 kg per leg). The exercise protocol involved sequential lower limb movements: hip flexion to 60°, knee flexion to 90°, and ankle dorsiflexion to 90°. Each movement was held for 25 s and performed sequentially and alternately between the right and left legs, completing 10 repetitions per leg. Immediately after completing the exercises, participants were instructed to stand up, and their blood pressure was measured at minutes 1, 3, 5, and 10.

Group C (lower extremity exercise only): Participants received a lower extremity exercise intervention only.

Participants received the same structured lower extremity exercise protocol without prior water consumption intervention. Before starting the exercise, participants rested in a supine position for five minutes, and baseline blood pressure was measured. The exercise protocol was conducted individually under nurse supervision using the same standardized protocol as Group B. Immediately after completing the exercise session, participants stood upright, and blood pressure measurements were recorded at 1, 3, 5, and 10 min after standing.

Group D (water consumption only): Participants received a standardized water consumption intervention only. Each participant was instructed to consume at least 2000 mL of water within a 24‐h period. Water intake was standardized using identical 2‐L bottles with volume indicators and participant identification labels. Adequacy of intake was confirmed by direct monitoring and documentation by ward nurses before proceeding with orthostatic blood pressure assessment. Once adequate hydration of 2 L was confirmed, participants were instructed to lie in a supine position for five minutes. Blood pressure was then measured and recorded in the supine position. Afterward, participants were instructed to stand up, and their blood pressure was measured at 1, 3, 5, and 10 min post‐standing.

#### 2.4.1. Instrument and Tools, and Measurement of OH

The instrument used in this study included a digital sphygmomanometer (General Care LD 582), a certified medical device, a pair of ankle weights (3 kg (1.5 kg per Leg)) for lower extremity exercise, and a 2 L tumbler labeled with the respondent’s name. To help document the research results, the author uses a documentation sheet in the form of a Data entry worksheet or fill‐in form for all group interventions. Intervention fidelity was maintained through standardized operating procedures (SOPs), prior nurse training, standardized intervention equipment, and direct supervision during intervention delivery, including the lower extremity exercise protocol and orthostatic blood pressure measurement protocol. Due to the nature of the intervention, participant and intervention‐provider blinding was not feasible. OH was assessed by comparing blood pressure changes between the supine and standing positions. Blood pressure was measured in the supine position after five minutes of rest and subsequently at 1, 3, 5, and 10 min after standing. The primary outcomes were changes in SBP and DBP.

### 2.5. Statistical Analysis

Data were analyzed using the *R* statistical program. Descriptive statistics were used to summarize participant characteristics. Baseline comparability between groups was assessed using descriptive comparison. Repeated measures ANOVA was performed to evaluate changes in SBP and DBP over time across the four groups. Bonferroni post‐hoc analysis was conducted for pairwise comparisons when significant group effects were identified. A *p*‐value < 0.05 was considered statistically significant.

The results of the characteristics, mean, and standard deviation of SBP and DBP data for each group during the 5‐day intervention are presented in Table 1.

Table 1 presents the baseline demographic and clinical characteristics of participants across the four intervention groups, along with the mean changes in SBP and DBP during the five‐day intervention period. The mean age of participants ranged from 39.38 to 45.55 years, with no statistically significant difference between groups (*p* = 0.19), indicating relatively comparable age distribution across intervention and control groups. Gender distribution was also similar across groups, with male participants comprising approximately 55%–58% of each group (*p* = 0.99). Likewise, the prevalence of diabetes and BMI categories did not differ significantly between groups (*p* = 0.56 for both variables). Smoking prevalence differed significantly between groups (*p* = 0.01), indicating potential variation in smoking exposure across intervention groups. Baseline orthostatic SBP and DBP changes did not differ significantly between groups (SBP: *p* = 0.09; DBP: *p* = 0.07), indicating relatively comparable baseline hemodynamic status prior to intervention. However, the distribution of antipsychotic treatment categories, including typical, atypical, and combination antipsychotic regimens, did not differ significantly between groups (*p* = 0.72), suggesting relatively comparable pharmacological exposure across the study population.

**TABLE 1:** Baseline demographic and clinical characteristics and mean changes in systolic and diastolic blood pressure across intervention groups.

Characteristic	Control	Combination	Exercise	Water	*p* Value
	45	45	45	45	
Male (%)	55.56	55.56	57.78	57.78	0.99
Diabetes (%)	15.5	11.11	8.9	6.7	0.56
Smoking (%)	57.78	26.67	55.56	73.33	0.01
BMI (%) Normal	71.1	64.4	62.2	68.9	0.56
Antipsychotic (%)					
Typical	6.67	11.11	6.67	4.44	0.72
Atypical	77.78	82.22	80.00	86.67	
Combination—typical and atypical	15.56	6.67	13.33	8.89	
Duration of drug					
Less than 1 year	42.2	40	44.4	37.8	0.92
More than 1 year	57.8	60	55.6	62.2	

**(Mean ± SD)**					

Age (mean ± SD)	45.55 ± 14.29	41.42 ± 14.37	39.71 ± 15.34	39.38 ± 14.20	0.19

SBP, mmHg (mean ± SD)	**(Mean ± SD)**				

Baseline SBP change	−12.07 ± 15.91	−10.89 ± 14.14	−8.34 ± 13.19	−10.82 ± 13.50	0.09
Day 1	−6.92 ± 13.09	−6.82 ± 10.41	−4.27 ± 11.98	−3.48 ± 12.45	
Day 2	−4.29 ± 14.66	−4.92 ± 10.88	−2.00 ± 11.77	−2.03 ± 10.73	
Day 3	−4.87 ± 12.80	−2.15 ± 13.52	−2.32 ± 12.08	−2.61 ± 10.04	
Day 4	−3.26 ± 12.14	−2.98 ± 9.75	−1.47 ± 9.63	−1.15 ± 11.94	
Day 5	−6.57 ± 10.97	−1.29 ± 11.20	−0.59 ± 9.88	−1.57 ± 10.84	

DBP, mmHg	**(Mean ± SD)**				

Baseline DBP change	−2.98 ± 9.29	−1.99 ± 7.48	−3.93 ± 10.31	−4.39 ± 9.98	0.07
Day 1	−3.00 ± 11.01	0.64 ± 8.47	1.21 ± 8.85	0.63 ± 9.49	
Day 2	0.71 ± 10.90	2.67 ± 8.35	3.37 ± 7.91	2.31 ± 7.79	
Day 3	−1.36 ± 11.01	5.34 ± 8.16	3.94 ± 10.69	2.34 ± 10.03	
Day 4	−1.13 ± 11.14	3.37 ± 8.07	2.03 ± 8.46	3.99 ± 9.80	
Day 5	0.21 ± 9.45	4.64 ± 7.71	0.74 ± 8.20	1.04 ± 8.40	

These findings in Table 2 indicate a clear preference for atypical antipsychotics, either as monotherapy or in combination, reflecting current clinical trends in psychiatric settings. The low prevalence of typical antipsychotic use, either alone or in combination, may be attributed to their higher risk of extrapyramidal side effects and cardiovascular complications, including OH. Most participants received atypical antipsychotic regimens, particularly combinations containing clozapine and risperidone, followed by aripiprazole combined with clozapine. The clozapine‐containing regimens are known to have a higher risk of OH due to alpha‐1 adrenergic blockade and autonomic dysfunction. Therefore, variation in antipsychotic regimens may have influenced orthostatic blood pressure responses in this study.

**TABLE 2:** Distribution of antipsychotic medication in schizophrenia with OH.

No	Category	*n* (%)
1	Mono typical antipsychotic	6.1
2	Mono atypical antipsychotic	42.2
3	Combination of typical antipsychotics	1.1
4	Combination of atypical antipsychotics	38.9
5	Combination of typical and atypical antipsychotics	11.7
	Total	100

The daily trends in mean SBP and DBP are shown in the figure below.

Figure 2 illustrates that, overall, the intervention groups demonstrated smaller orthostatic SBP reductions compared with the control group over the five‐day intervention period. The combined intervention group (leg exercise and water consumption) and the leg exercise group showed relatively more stable SBP patterns across repeated standing measurements, whereas the control group exhibited greater fluctuations and larger decreases in SBP, particularly on Days 3 and 5. Although the temporal trend did not reach statistical significance, the intervention effect was statistically significant, indicating that the interventions contributed to improved SBP stabilization compared with routine ward care alone.

**FIGURE 2:**
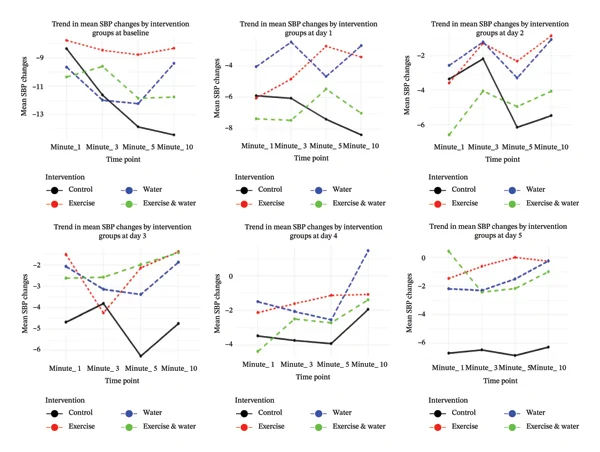
Daily trends in mean systolic blood pressure (SBP) changes across intervention groups from baseline to day 5.

As shown in Figure 3, overall, the intervention groups demonstrated more stable and improved DBP responses compared with the control group throughout the intervention period. The combined intervention group (leg exercise and water consumption) consistently showed higher positive DBP changes across repeated standing measurements, particularly on Days 3 to 5, indicating improved hemodynamic stabilization following postural changes. In contrast, the control group continued to demonstrate greater fluctuations and persistent reductions in DBP values.

**FIGURE 3:**
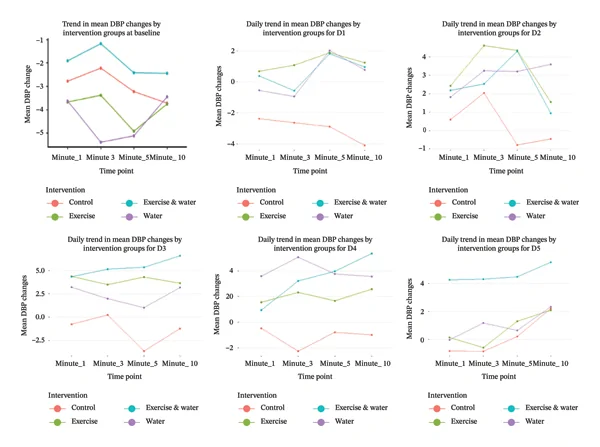
Daily trends in mean diastolic blood pressure (DBP) changes across intervention groups from baseline to day 5.

## 3. Results

The final analysis included 180 participants across the four intervention groups. Participants who did not complete the intervention protocol or had incomplete blood pressure measurements during follow‐up were not included in the final analysis. Therefore, all participants included in the analysis completed the 5‐day follow‐up and had complete outcome data. Table 1 presents the demographic characteristics of each group, along with the mean and standard deviation of SBP and DBP from Day 1 to Day 5. Baseline mean SBP and DBP changes did not differ significantly between groups (SBP: *p* = 0.09; DBP: *p* = 0.07), indicating relatively comparable baseline hemodynamic status prior to intervention. Study result of the daily trends in mean SBP changes across intervention groups from baseline to Day 5. Overall, the intervention groups demonstrated more stable SBP responses compared with the control group throughout the intervention period. Meanwhile, the daily trends in mean DBP change across intervention groups from baseline to Day 5. The combined intervention group demonstrated more favorable DBP stabilization patterns compared with the control group over the five‐day intervention period.

The results of the repeated measures ANOVA (RM ANOVA) for SBP on Day 5 revealed a statistically significant difference between the intervention groups (F (3176) = 4.322, *p* = 0.006), with a small to moderate effect size (generalized. *η* ^2^ = 0.047). However, no significant main effect of time (*p* = 0.597) or intervention by time interaction (*p* = 0.716) was observed. Estimated marginal means (EMM) indicated that the lower extremity exercise group had the highest adjusted mean SBP improvement (EMM = 0.00 mmHg, 95% CI‐3.37 to 3.37), followed by the hydration group (EMM = −1.51 mmHg), the combined exercise and hydration group (EMM = −2.18 mmHg), and the control group (EMM = −6.87 mmHg). Bonferroni‐adjusted pairwise comparisons demonstrated a significant difference only between the lower extremity exercise and control groups (mean difference = −6.87 mmHg, 95% CI‐13.32 to −0.42, *p* = 0.030), whereas no statistically significant differences were observed between the remaining intervention groups.

Similarly, RM ANOVA for DBP revealed a significant main effect of intervention (F (3,176) = 3.895, *p* = 0.010), with a small to moderate effect size (generalized. *η* ^2^ = 0.042). A statistically significant main effect of time was also observed (F (3,528) = 5.223, *p* = 0.001). However, no significant interaction effect between intervention and time was identified (*p* = 0.922). EMM indicated that the combined lower extremity exercise and hydration group had the highest adjusted mean DBP improvement in DBP (EMM = 4.47 mmHg, 95% CI 1.93 to 7.00), followed by the lower extremity exercise group (EMM = 1.31 mmHg, 95% CI−1.22 to 3.85), the hydration group (EMM = 0.67 mmHg, 95% CI−1.87 to 3.20), and the control group (EMM = 0.22 mmHg, 95% CI−2.31 to 2.76). Bonferroni‐adjusted pairwise comparisons showed no statistically significant differences between any intervention groups after adjustment (all *p* > 0.05), although the combined lower extremity exercise and hydration group had the highest estimated marginal mean for DBP.

## 4. Discussion

This study examined the effectiveness of lower extremity exercise, water consumption, and their combination in reducing OH among patients with schizophrenia receiving antipsychotic therapy. This study provides several novel contributions to the existing evidence on nonpharmacological management of OH. First, unlike previous intervention studies that primarily evaluated a single intervention in older adults or neurological populations, this study directly compared lower extremity exercise, water consumption, and their combination within the same psychiatric inpatient population. Second, patients with schizophrenia represent a unique clinical population because OH is influenced not only by autonomic dysfunction associated with antipsychotic therapy but also by sedentary behavior, reduced physical activity, and inconsistent fluid intake during hospitalization. Finally, by repeatedly evaluating blood pressure responses over five consecutive days, this study provides evidence regarding the short‐term trajectory of hemodynamic recovery following nurse‐led nonpharmacological interventions, thereby extending previous evidence that has largely focused on immediate or single‐session effects.

The findings demonstrated that all intervention strategies significantly improved SBP compared with routine care, while the combined intervention showed the highest estimated marginal mean for DBP improvement. These findings suggest that nonpharmacological interventions can be integrated into psychiatric nursing care as practical strategies for reducing OH.

The improvement in SBP observed across all intervention groups may be explained by the physiological mechanisms of increased venous return and improved autonomic regulation. Lower extremity exercise activates the skeletal muscle pump, enhancing venous return and supporting cardiovascular adaptation during postural changes. Similarly, water consumption increases intravascular volume and improves hemodynamic stability, thereby reducing the risk of blood pressure decline upon standing. These findings are consistent with previous studies showing that both lower limb exercise and hydration and physical exercise can improve orthostatic tolerance [9, 11, 13, 15, 16].

The superior effect of the combined intervention on DBP may be attributed to the synergistic interaction between plasma volume expansion and increased systemic vascular resistance. DBP is strongly influenced by vascular tone, and the combination of hydration and muscle pump activation may provide more effective stabilization of vascular resistance during orthostatic challenge. Previous studies have similarly demonstrated that acute water intake can stimulate sympathetic nervous system activity and increase vascular resistance, while lower limb exercise improves venous return and baroreflex responsiveness [14, 17, 18]. Increasing intravascular volume by enhancing daily fluid intake to 2 L and/or dietary salt consumption (6–10 g) has been shown to be an effective strategy in the prevention of OH [19]. Simple behavioral strategies such as physical counter‐pressure maneuvers, including leg crossing, squatting, stooping, and isometric contraction of the leg, abdominal, and gluteal muscles, have also been recommended for patients with OH to improve orthostatic tolerance [1, 2, 6, 12, 20, 21]. Other reviews have also emphasized the role of nonpharmacological management strategies, including fluid loading, compression garments, and gradual positional changes [22, 23]. Previous literature has emphasized adequate hydration as an important management strategy for antipsychotic‐induced OH [5], which is consistent with the findings of the present study. By addressing the underlying physiological mechanisms, decreased vascular tone and impaired baroreflex function, our nonpharmacological interventions serve as practical nursing strategies to enhance patient safety and treatment adherence.

These findings are particularly relevant in psychiatric inpatient populations, where patients often experience medication‐induced autonomic dysfunction, physical inactivity, and inconsistent fluid intake. Antipsychotic medications, particularly those with alpha‐1 adrenergic receptor blockade, impair vasoconstrictive responses during postural changes, increasing the risk of OH. In this study, most participants were prescribed atypical antipsychotics, particularly risperidone and clozapine, both of which are known to contribute to OH through autonomic dysregulation [5, 24, 25]. Antipsychotic medications exert effects on alpha‐1 adrenergic receptors in peripheral blood vessels, impairing vasoconstrictive responses during postural changes and contributing to OH [26]. Therefore, findings from studies conducted in older adults or patients with neurological disorders cannot be directly generalized to psychiatric inpatients receiving antipsychotic therapy, highlighting the importance of population‐specific evidence.

Although the present study demonstrated significant improvements following lower extremity exercise and water consumption, previous evidence regarding nonpharmacological management of OH has not been entirely consistent. Systematic reviews have reported heterogeneous findings, with several interventions demonstrating only modest or nonsignificant effects because of differences in patient populations, intervention protocols, outcome measures, and generally small sample sizes. Reference [12] reported mixed effectiveness across nonpharmacological interventions, while [27] concluded that the overall evidence remains limited due to substantial methodological heterogeneity. Likewise, [28] highlighted that many commonly recommended interventions are supported by only limited evidence.

Antipsychotic‐induced OH has consistently been associated with increased fall risk, impaired balance, and cardiovascular instability among psychiatric patients [7, 8, 29–31]. These findings reinforce the importance of preventive nursing interventions to improve patient safety in psychiatric inpatient settings. Clozapine, in particular, has been associated with several serious side effects, including myocarditis, OH, and tachycardia. Reference [32] also reported OH as a notable adverse effect of antipsychotic therapy. Considering these risks, nursing interventions that aim to enhance physical activity and prevent OH are crucial, as they not only promote hemodynamic stability but also reduce the likelihood of falls, thereby improving overall patient safety and outcomes [5].

By targeting the physiological mechanisms involved, namely, reduced vascular tone and impaired baroreflex function, our combined intervention presents a viable and practical nursing strategy to enhance patient safety, improve adherence to antipsychotic treatment, and reduce the risk of falls in psychiatric settings. Nurses’ involvement in preventive and educational efforts for patients is crucial both during and after treatment. Haruna et al. and Lourenço et al. [33, 34] stated that nursing training is necessary for early clinical deterioration risk assessment based on protocols for nurses, including recognizing patient vital signs.

From a nursing perspective, these findings support the implementation of structured nonpharmacological interventions as part of routine psychiatric inpatient care. Lower extremity exercise and hydration are simple, low‐cost, and feasible interventions that can be incorporated into nursing protocols to reduce OH, improve cardiovascular stability, and reduce fall risk. Early identification of OH and timely nursing intervention may improve patient safety and treatment adherence. Furthermore, integrating structured hydration and lower extremity exercise protocols into routine psychiatric nursing care may contribute to improved hemodynamic stability, reduced fall risk, and enhanced patient safety among individuals receiving antipsychotic treatment.

### 4.1. Strengths of the Study

Several methodological and clinical strengths enhance the robustness and practical relevance of the present study. To our knowledge, this is one of the first quasiexperimental studies to compare the effectiveness of lower extremity exercise, water consumption, and their combination for the management of OH among hospitalized patients with schizophrenia receiving antipsychotic treatment. The study included a relatively large sample of 180 participants equally distributed across four intervention groups, providing adequate statistical power to compare the interventions.

Another strength is the use of standardized intervention protocols and repeated orthostatic blood pressure measurements at multiple time points over five consecutive days. This approach allowed a comprehensive evaluation of both SBP and DBP responses during postural changes while enhancing intervention fidelity and measurement consistency. Furthermore, the interventions evaluated in this study are simple, low‐cost, nonpharmacological, and nurse‐led strategies that can be readily incorporated into routine psychiatric inpatient care, thereby increasing the clinical applicability of the findings.

### 4.2. Study Limitations

Despite the encouraging findings, this study has several limitations. Ward‐based nonrandomized allocation may have introduced potential selection bias despite efforts to maintain baseline comparability between groups. Because the interventions involved supervised lower extremity exercise and hydration, blinding of participants and outcome assessors was not feasible, which may have introduced performance and detection bias. Smoking prevalence differed significantly across groups, and antipsychotic medication dosages were not controlled as covariates. Furthermore, the intervention period was limited to five consecutive days, preventing evaluation of the long‐term sustainability of blood pressure improvement and clinical outcomes. Despite these limitations, this study provides practical evidence supporting nurse‐led, low‐cost nonpharmacological interventions for the management of OH in psychiatric inpatient settings.

## 5. Conclusion

This study demonstrated that nonpharmacological interventions, specifically lower extremity exercise and water consumption, are effective in reducing OH among patients with schizophrenia receiving antipsychotic therapy.

These findings support the use of simple, low‐cost, and feasible nursing interventions to mitigate the risk of falls, improve cardiovascular stability, and enhance the overall safety of psychiatric treatment. Implementing these interventions into routine psychiatric nursing care may offer a practical strategy to reduce morbidity related to antipsychotic‐induced OH.

## 6. Recommendations

In light of the findings from this study, several evidence‐informed recommendations are proposed to enhance both clinical practice and future research directions. Firstly, psychiatric nurses should be equipped through structured training programs to incorporate nonpharmacological interventions, particularly lower extremity exercises and water consumption, into routine nursing care for patients identified as being at risk for OH. These strategies offer safe, low‐cost alternatives to pharmacological approaches and may be feasibly implemented within inpatient mental health settings. Secondly, institutional‐level policy development is essential. Mental health care facilities should establish standardized protocols or clinical guidelines to ensure consistent implementation of nonpharmacological management for OH, fostering both patient safety and quality of care. Thirdly, longitudinal studies are strongly recommended to assess the sustained impact of such interventions on orthostatic symptomatology, fall prevention, adherence to treatment, and health‐related quality of life. Lastly, future investigations should broaden their scope to include vulnerable populations, particularly elderly patients with psychiatric disorders, who may experience compounded risks due to age‐related autonomic dysfunction and psychotropic medication use.

## Funding

This study received no funding from any organization or individual.

## Ethics Statement

This study has received ethical approval from the Health Research Ethics Committee of University of Catholic Atmajaya, No. 0006P/III/PPPE.PM.10.05/06/2023.

## Conflicts of Interest

The authors declare no conflicts of interest.

## Data Availability

The dataset generated and analyzed during this study has been deposited in the Zenodo repository and is publicly available at https://doi.org/10.5281/zenodo.17636040. All data have been fully de‐identified to protect participant confidentiality (Narulita, S., 2025).
